# Adherence to BNF Prescribing Standards and PRN Governance in an Early Intervention in Psychosis Service: TwoCycle Clinical Audit

**DOI:** 10.1192/bjo.2026.11653

**Published:** 2026-06-30

**Authors:** Sita Asi, Yassmine Sabtan, Nandini Chakraborty, Muhammad Ahmed, Dina Elamien

**Affiliations:** 1 LPT, Leicester, United Kingdom; 2 University of East Anglia, Norwich, United Kingdom; 3 UHL, Leicester, UAE

## Abstract

**Aims:**

(1) Quantify adherence to British National Formulary (BNF) dose limits; (2) Measure polypharmacy prevalence and rationale documentation; (3) Assess as required (PRN) governance (instruction clarity, review frequency, benzodiazepine duration); and (4) Compare high-dose antipsychotic therapy (HDAT) form completion between cycles.

**Methods:**

Retrospective analysis of electronic health records (EHR) exported to standardised spreadsheets. Polypharmacy defined as being more than 2 medications of the same class. Doses were expressed as %BNF maximum across six medication classes: >100% denoted high-dose prescribing. PRN indicators captured instruction presence, review status, and benzodiazepine duration ≤4 weeks. Analyses were performed programmatically on two audit cycles.

**Results:**

BNF adherence worsened: patients exceeding BNF maxima decreased from **1%** (Cycle 1) to **2.9%** (Cycle 2).

Polypharmacy increased from **5.6%** to **10.4%**; (e.g., switching medications/complex illness/patient preference) likely contributed, but the rise suggests persistent clinical complexity.

PRN governance was mixed: “PRN not reviewed” improved from **1.9%** to **1.45%**, while “PRN instruction missing” rose from **1.1%** to **2.1%**.

Benzodiazepine PRN >4 weeks increased from **0.26%** to **2.70%**, indicating a stewardship gap.

HDAT documentation remained suboptimal but improved: **1/378 (0.002%)** forms completed in Cycle 1 vs **3/482 (0.62%)** in Cycle 2.

**Conclusion:**

The re-audit demonstrates **worsening of BNF dose exceedance** and a **modest improvement in PRN review**, evidencing strengthened prescribing safety processes. Nevertheless, **polypharmacy increased,** and **benzodiazepine PRN durations** lengthened, while **HDAT form completion** remains low. Priorities are: (i) embedding EHR “hard-stops” for >100% BNF doses and missing PRN instructions; (ii) automated four-week PRN benzodiazepine review prompts with default taper plans; (iii) a structured polypharmacy pro-forma recording indication, expected benefit, and review date; and (iv) targeted HDAT training plus mandatory electronic prompts for form completion. A third-cycle re-audit using identical denominators is recommended to verify sustained improvement and close remaining governance gaps

